# Willingness and beliefs associated with reporting travel history to high-risk coronavirus disease 2019 epidemic regions among the Chinese public: a cross-sectional study

**DOI:** 10.1186/s12889-020-09282-4

**Published:** 2020-07-25

**Authors:** Lifeng Wei, Zhuowa Sha, Ying Wang, Gangyu Zhang, Haonan Jia, Shuang Zhou, Yuanheng Li, Yameng Wang, Chao Liu, Mingli Jiao, Shufan Sun, Qunhong Wu

**Affiliations:** 1 Harbin Medical University, 157 Baojian Road, Nangang District, Harbin, 150086 Heilongjiang China; 2 Heilongjiang Provincial Psychiatric Institute, Xiangfang District, Harbin, Heilongjiang China

**Keywords:** Coronavirus disease 2019 (COVID-19), Travel history report, Withholding

## Abstract

**Background:**

The Coronavirus Disease 2019 (COVID-19) that first occurred in Wuhan, China, is currently spreading throughout China. The majority of infected patients either traveled to Wuhan or came into contact with an infected person from Wuhan. Investigating members of the public with a travel history to Wuhan became the primary focus of the Chinese government’s epidemic prevention and control measures, but several instances of withheld histories were uncovered as localized clusters of infections broke out. This study investigated the public’s willingness and beliefs associated with reporting travel history to high-risk epidemic regions, to provide effective suggestions and measures for encouraging travel reporting.

**Methods:**

A cross-sectional study was conducted online between February 12 and 19, 2020. Descriptive analysis, chi-squared test, and Fisher’s exact test were used to identify socio-demographic factors and beliefs associated with reporting, as well as their impact on the willingness to report on travel history to high-risk epidemic regions.

**Results:**

Of the 1344 respondents, 91 (6.77%) expressed an inclination to deliberately withhold travel history. Those who understood the benefits of reporting and the legal consequences for deliberately withholding information, showed greater willingness to report their history (*P* < 0.05); conversely, those who believed reporting would stigmatize them and feared being quarantined after reporting showed less willingness to report (*P* < 0.05).

**Conclusions:**

As any incident of withheld history can have unpredictable outcomes, the proportion of people who deliberately withhold information deserves attention. Appropriate public risk communication and public advocacy strategies should be implemented to strengthen the understanding that reporting on travel history facilitates infection screening and prompt treatment, and to decrease the fear of potentially becoming quarantined after reporting. Additionally, social support and policies should be established, and measures should be taken to alleviate stigmatization and discrimination against potential patients and reporters of travel history. Reinforcing the legal accountability of withholding travel history and strengthening systematic community monitoring are the measures that China is currently taking to encourage reporting on travel history to high-risk epidemic regions. These non-pharmaceutical interventions are relevant for countries that are currently facing the spread of the epidemic and those at risk of its potential spread.

## Introduction

Since the emergence of the first case of coronavirus disease (COVID-19) infection on December 31, 2019, the Severe Acute Respiratory Syndrome Coronavirus 2 (SARS-CoV-2) has spread rapidly throughout China [1]. There is no strong evidence that the coronavirus originated in China, but the epidemic outbreak in China was found to exist in an epidemiological link to the wholesale seafood market in Wuhan, China [2]. By March 26, 2020, the number of confirmed cases in the city of Wuhan had reached 50,006 people, and accounted for 61.48% of all confirmed cases in the country [3]. This made Wuhan a high-risk region for the epidemic in China. Nearly all of the infected patients emerging from other regions had some sort of connection with Wuhan, either by having traveled to Wuhan, or by coming into contact with infected patients from Wuhan [4].

The Chinese government implemented appropriate and timely response measures to contain the outbreak, by quarantining Wuhan City and setting up inspection points in other regions for people with a travel history to Wuhan or its surrounding regions [2]. This method of inspection relies on the public to submit reports voluntarily, but its effectiveness does not appear to be satisfactory. Several cases of withheld information were uncovered subsequent to the onset of an individual’s illness or an outbreak of COVID-19 in localized clusters. For example, a man from Jinjiang, Fujian, lied that he was returning from the Philippines when he was in fact traveling home from Wuhan. He carried out regular activities and attended gatherings, which resulted in the home quarantine of over 4000 people. Another individual from Ya’an, Sichuan, became ill after returning from Wuhan. Despite being repeatedly questioned by medical staff whether they had visited Wuhan or its surrounding regions, Hou never admitted the truth. Exposure to this individual resulted in the quarantine of over 100 people, including more than 30 medical staff. Another person in Hebei, who withheld their history died after missing the opportune time for treatment, leading to 77 close contacts being quarantined. Tens of thousands of people have been forced to undergo quarantine and to shoulder the risk of infection, resulting in a devastating outcome and consequences that still cannot be properly estimated.

The act of withholding travel history to high-risk regions is posing a difficult challenge for epidemic prevention and control, and has greatly undermined epidemic response measures. Worse still, the epidemic is no longer limited to China. More than a hundred countries including the United States, Italy, and the United Kingdom have reported cases of the infection, and the number of afflicted countries and cases continues to rise. A study on the public’s willingness to report on travel history to high-risk epidemic regions will provide support for countries that are facing or will possibly face the epidemic. Therefore, this study’s goals were as follows: 1) to elucidate and analyze the public’s willingness and beliefs associated with reporting travel history to high-risk epidemic regions; 2) to provide effective suggestions and measures for encouraging the public to report their travel history in response to the World Health Organization’s (WHO) advocacy for non-pharmaceutical interventions.

## Methods

### Questionnaire design

After conducting the necessary literature research and receiving expert advice, we developed a self-administered questionnaire (Additional file 1). To confirm the effectiveness of the questionnaire, we invited seven experts from fields such as health emergency, epidemiology, and public psychology, as well as three health administration workers who are currently in charge of epidemic prevention and control, to engage in two rounds of reviews of the questionnaire. In addition, we formed a team of 30 people to carry out a two-week reliability re-test (Cronbach’s α = 0.75). The team was recruited voluntarily from Harbin, Heilongjiang Province, China, based on the principle of convenience. They responded to the entire questionnaire and again two weeks later. The data obtained was not used for the final analysis. This questionnaire included the following parts: (1) Socio-demographic information; (2) Willingness and beliefs associated with reporting on travel history to high-risk epidemic regions; (3) Cues that promote reporting on travel history to high-risk epidemic regions.

The dependent variable in this study was the respondents’ self-rated willingness to report on travel history to high-risk regions, which was evaluated by the item: ‘Supposing you have a travel history to Wuhan, would you report this to the designated department, facility, or personnel?’ using a five-point Likert scale ranging from 1 to 5 (definitely would not report, would not report, unsure, would report, definitely would report). During analysis, for the purpose of comparing respondents who had a positive willingness toward reporting with those who did not, we sorted those who selected 4 and 5 into the category ‘report’, and those who selected 1, 2, and 3 into the category ‘not report’. In the results of this study, the number of respondents who chose each item of the dependent variable was as follows: 1–5(0.37%), 2–13(0.97%), 3–73(5.43%), 4–107(7.96%), and 5–1146(85.27%).

Socio-demographic data were collected, including the respondents’ gender, age, education level, marital status, place of residence, living arrangement, and religious belief. Among them, the religious belief data was obtained by the item: ‘Do you have a religion’, using Yes or NO as the answer option. In addition, we used the MacArthur Scale of Subjective Social Status to evaluate the respondents’ social status. This scale yields comprehensive ratings for the level of income, occupation, and education [5]. The scale contains two items; respondents rated their perceived social status within their country and community from 1 (the bottom) to 10 (the top), with the total points falling within a range of between 2 and 20. Respondents were divided into two groups according to the median: ≤ 11 was lower class; > 11 was upper class.

The respondents’ beliefs associated with reporting on travel history was assessed through nine prompts, to which they indicated their degree of agreement by selecting a rating between 1 (strongly disagree) and 5 (strongly agree). The respondents’ overall perceptions of travel history and the COVID-19 infection were evaluated by the following prompt: ‘Travel history has an impact on contracting COVID-19’. The respondents’ perceptions of the benefits of reporting were assessed by two prompts: ‘Reporting can confirm whether I contracted COVID-19 earlier’ and ‘Reporting can help discover earlier potential patients infected by coming in contact with me’. The respondents’ perceptions of obstacles to reporting were assessed by five prompts, including ‘Reporting makes me feel stigma’. In addition, another prompt judged respondents’ perception of the consequences of withholding travel history: ‘Withholding travel history to high-risk epidemic regions will result in legal liability.’ During analysis, all responses to the prompts were converted into binary variables: respondents who rated 4 and 5 were sorted into the category ‘agree’, whereas respondents who rated 1, 2, and 3 were sorted into the category ‘disagree’.

In the questionnaire, we also designed an item to investigate which cues would promote respondents to report. The item allowed multiple selections, including cues such as ‘persuasion by family or friends’, ‘community public speaking (via loudspeaker, bulletin board, etc.)’, and ‘people around me showing potential symptoms of COVID-19’.

### Sample and data collection

The cross-sectional study was carried out in the format of an anonymous web-based questionnaire. The questionnaire survey was conducted among residents of nine provinces with varying epidemic levels, selected based on the cumulative data of confirmed COVID-19 cases publicized by the National Health Commission of China, the health commissions of various provinces, cities, and districts, and the governments of each province, city, and district, as of February 11, 2020. Provinces where the epidemic was more severe were Henan, Zhejiang, and Guangdong (cumulative confirmed cases, ≥ 1000 people); provinces where the epidemic was moderately severe were Heilongjiang, Anhui, and Yunnan (cumulative confirmed cases, 100–999 people); provinces where the epidemic was less severe were Jilin, Inner Mongolia, and Gansu (cumulative confirmed cases, ≤ 99 people). Based on the ranked cumulative numbers of confirmed cases of the cities within each province, a city where the epidemic was severe (cumulative number of confirmed cases exceeding the mean value of all cities in the province) and a city where the epidemic was relatively mild (cumulative number of confirmed cases being lower than the mean value of all cities in the province) were drawn from each province.

Using the method of convenience sampling, questionnaire administrators were recruited from the sample cities, who were undergraduates or postgraduates of Harbin Medical University and postponed their return to school due to COVID-19. In addition to recruiting two administrators in the three sample cities, all the other sample cities recruited an administrator, for a total of 21 administrators. They sent a link to the questionnaire to the residents in their communities through social networking software such as WeChat (WeChat is the largest social media in China similar to Facebook and Twitter, and the number of monthly active accounts has reached to 1.165 billion [6]), alongside an explanation of the study’s intention and anonymity. Those who received the link could voluntarily decide whether to respond, and we required at least 100 people in each sample city to receive the link. The data were collected between February 12 and 19, 2020. Finally, a total of 1965 people received the link. In addition, according to the questionnaire network platform, 1823 people clicked on the link and 1481 people responded to the questionnaire (potential response rate = 75.37%). The researchers carefully reviewed the questionnaires and eliminated those submitted by participants under the age of 18 years and participants who selected the same answers to every question. A total of 1344 valid questionnaires were finally collected (effective response rate = 90.75%).

### Data analysis

Descriptive analysis was used to show the socio-demographic characteristics of the respondents, their willingness and beliefs associated with reporting on travel history to high-risk regions, and cues that could potentially promote reports. Chi-squared test and Fisher’s exact test were used to analyze the correlation between the participants’ willingness and beliefs to report. SPSS 22.0 (IBM, 2010) was used to conduct the analysis. Statistical significance was set at *P* < 0.05.

## Results

### Characteristics of the respondents

More than half of the respondents (60.19%) were women. The ages of the respondents were concentrated between 21 and 30 years old (37.80%). Among the respondents, 61.76% had an education level of undergraduate or above, and 56.03% were single. More than half of the respondents (74.48%) normally resided in cities, and a majority lived with family or friends (89.73%). Almost all of the respondents (90.10%) were non-religious. The majority of the respondents (74.33%) perceived themselves to be lower class in social status.

6.77% of the respondents chose not to report when they had a travel history to Wuhan, and 93.23% of the respondents chose to report. Gender, age, educational level, marital status, place of residence, and living arrangement were significantly correlated to willingness to report on travel history (Table 1). 9.35% of men chose not to report, which was higher than that of women (5.07%). Respondents under the age of 20 had the highest non-reporting rate (12.79%), while those aged 41 to 50 had the lowest rate (1.92%). In terms of education level, the proportion of people who chose not to report was the highest among college graduates (9.33%), but the lowest among those with master degree and above (3.01%). Single respondents had a higher percentage of non-reporter (9.56%) than married and widowed/separated/divorced. In addition, about one-tenth of the respondents who live in countryside (10.20%) chose not to report, which was higher than that of those who live in city (5.59%). The proportion of people who chose not to report among the respondents living with others (28.57%) was higher than living alone (7.69%) and living with family or friends (6.30%).

**Table 1:** Socio-demographic characteristics of respondents (*n* = 1344)

Characteristics	Total N (%)	Willingness to report travel history to high-risk epidemic regions		*p*-value
		Not report (*n* = 91)	Report (*n* = 1253)	
Gender				0.002^**^
Male	535 (39.81)	50 (9.35)	485 (90.65)	
Female	809 (60.19)	41 (5.07)	768 (94.93)	
Age				0.000^**^
≤ 20	258 (19.20)	33 (12.79)	225 (87.21)	
21–30	508 (37.80)	42 (8.27)	466 (91.73)	
31–40	143 (10.64)	6 (4.20)	137 (95.80)	
41–50	261 (19.42)	5 (1.92)	256 (98.08)	
≥ 51	174 (12.95)	5 (2.87)	169 (97.13)	
Education level				0.001^**^
High school graduate and below	336 (25.00)	12 (3.57)	324 (96.43)	
Junior college	178 (13.24)	10 (5.62)	168 (94.38)	
College graduate	697 (51.86)	65 (9.33)	632 (90.67)	
Master degree and above	133 (9.90)	4 (3.01)	129 (96.99)	
Marital status				0.000^**^
Single	753 (56.03)	72 (9.56)	681 (90.44)	
Married	546 (40.63)	15 (2.75)	531 (97.25)	
Widowed/Separated/Divorced	45 (3.35)	4 (8.89)	41 (91.11)	
Place of residence				0.003^**^
City	1001 (74.48)	56 (5.59)	945 (94.41)	
Countryside	343 (25.52)	35 (10.20)	308 (89.80)	
Living arrangement				0.003^**^
Living alone	117 (8.71)	9 (7.69)	108 (92.31)	
Living with family or friends	1206 (89.73)	76 (6.30)	1130 (93.70)	
Living with others	21 (1.56)	6 (28.57)	15 (71.43)	
Religious belief				0.468
No religion	1211 (90.10)	80 (6.61)	1131 (93.39)	
Religious	133 (9.90)	11 (8.27)	122 (91.73)	
Subjective social status				0.683
Lower-class	999 (74.33)	66 (6.61)	933 (93.39)	
Upper-class	345 (25.67)	25 (7.25)	320 (92.75)	

**P < 0.01; *P < 0.05

### Beliefs associated with reporting on travel history to high-risk epidemic regions

Overall, 83.04% of respondents believed that having a travel history to high-risk epidemic regions would increase chances of contracting COVID-19. Over four out of five respondents agreed that reporting on travel history can help to confirm whether they had contracted the infection sooner (83.26%), and discover potential patients who were infected by coming in contact with them earlier (87.13%). About half (57.44%) of the respondents believed that reporting would make them feel stigmatized. Other percentages of respondents believed reporting would result in high follow-up checks or treatment expenses (13.84%), did not know how to report (17.34%), and believed reporting was very inconvenient (8.26%). In addition, 5.88% of the respondents expressed fear of potential quarantine after reporting on their travel history. Overall, nearly one-fourth of the respondents (23.96%) did not believe withholding information on travel history to high-risk regions would result in legal liability. Table 2 displays the factors that are significantly correlated with a willingness to report on travel history to high-risk epidemic regions.

**Table 2:** Beliefs associated with reporting travel history to high-risk epidemic regions (*n* = 1344)

	Total N (%)	Willingness to report travel history to high-risk epidemic regions		*p*-value
		Not report (n = 91)	Report (n = 1253)	
Travel history has an impact on contracting COVID-19				0.199
Disagree	228 (16.96)	11 (4.82)	217 (95.18)	
Agree	1116 (83.04)	80 (7.17)	1036 (92.83)	
Reporting can confirm whether I contracted COVID-19 earlier				0.000^**^
Disagree	225 (16.74)	67 (29.78)	158 (70.22)	
Agree	1119 (83.26)	24 (2.14)	1095 (97.86)	
Reporting can help discover earlier potential patients infected by coming in contact with me				0.000^**^
Disagree	173 (12.87)	64 (36.99)	109 (63.01)	
Agree	1171 (87.13)	27 (2.31)	1144 (97.69)	
Reporting makes me feel stigma				0.000^**^
Disagree	572 (42.56)	59 (10.31)	513 (89.69)	
Agree	772 (57.44)	32 (4.15)	740 (95.85)	
Reporting would lead to great expenses for follow-up tests or treatment				0.658
Disagree	1158 (86.16)	77 (6.65)	1081 (93.35)	
Agree	186 (13.84)	14 (7.53)	172 (92.47)	
I don’t know how to submit a report				0.355
Disagree	1111 (82.66)	72 (6.48)	1039 (93.52)	
Agree	233 (17.34)	19 (8.15)	214 (91.85)	
Reporting is very inconvenient				0.839
Disagree	1233 (91.74)	84 (6.81)	1149 (93.19)	
Agree	111 (8.26)	7 (6.31)	104 (93.69)	
I’m afraid that reporting may lead to my quarantine				0.000^**^
Disagree	1265 (94.12)	23 (1.82)	1242 (98.18)	
Agree	79 (5.88)	68 (86.08)	11 (13.92)	
Withholding travel history to high-risk epidemic regions will result in legal liability				0.000^**^
Disagree	322 (23.96)	70 (21.74)	252 (78.26)	
Agree	1022 (76.04)	21 (2.05)	1001 (97.95)	

**P < 0.01; *P < 0.05

### Cues that promote reports of travel history to high-risk epidemic regions

**Fig. 1:**
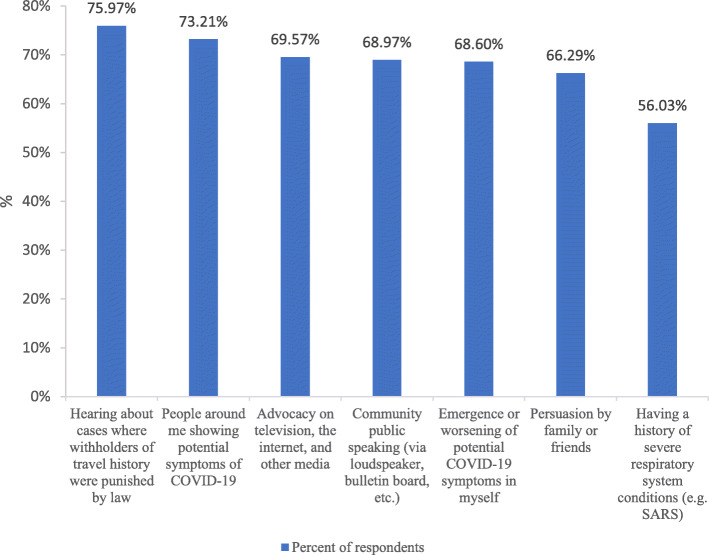
Cues promoting the reporting of travel history to high-risk epidemic regions

Figure 1 displays the cues that respondents perceived may encourage their reporting of travel history to high-risk epidemic regions. ‘Hearing about cases where withholders of travel history were punished by law’ was the most selected option (75.97%), followed by ‘people around me showing potential symptoms of COVID-19’ (73.21%), and ‘advocacy on television, the internet, and other media’ (69.57%).

## Discussion

Our study evaluated the current willingness and beliefs associated with reporting on travel history to high-risk epidemic regions among the Chinese public, as well as the correlation between their willingness and beliefs. A proportion of the public (6.77%) was found to be more inclined to withhold their history, although the majority agreed to report. Nonetheless, considering that any withheld history case can result in unpredictable outcomes, this result already deserves great attention [7–9]. After the emergence of frequent incidences of withheld history, Chinese authorities implemented more severe measures, including restricting population movement in cities and communities, rewarding those who provide clues to withholders, and instigating systematic blanket searches in communities [10, 11]. However, in all of these actions the public plays a relatively passive role. Therefore, research into the association between the public’s willingness and beliefs to report on travel history is essential for promoting the public’s voluntary reporting.

This study discovered several factors associated with the willingness to report on travel history to high-risk epidemic regions. First, is the belief of benefiting from reporting. Apart from being able to test whether one is infected as soon as possible, a more valuable benefit of timely reporting on travel history, is that it helps to identify other potentially infected patients. The department responsible for epidemic prevention and control would be able to investigate the public transportation that reporters took, the recent events in which they participated, and the number of people that they had been in contact with. This response would allow potentially infected patients to be examined and prevent them from further spreading the virus [12]. The results of the study reflect that the proportion of the public that recognized these benefits demonstrated a greater willingness to report (*P* < 0.05). However, our research cannot determine whether the reporting behavior of the public could be attributed to egoistic behavior or altruistic behavior. Emphasizing all the benefits of reporting in public advocacy may be the better option for promoting a willingness to report. Furthermore, prospect theory in behavioral economics asserts that people have a mentality of aversion toward loss; their level of sensitivity toward losses and gains are unequal, and the pain they feel in the face of loss is far greater than the joy of gaining [13]. Thus, instead of promoting ‘the benefits of reporting’, the government can promote ‘the risks of not reporting’ in public advocacy to activate the loss-averse mentality in the public and encourage action.

Second, considering the perceived obstacles to reporting, the feelings of stigmatization that reporting brings and the fear of potential quarantine are factors that influence the willingness to report. The proportion of the public that believed reporting would cause stigmatization and feared the quarantine that they may face after reporting expressed a lower level of willingness to report (*P* < 0.05). The stigmatization and discrimination of potential patients are phenomena that were present since the early stage of epidemic outbreak [14]. Reporting on travel history might cause the reporter to be labeled a potential patient, which lowers their willingness to report [15]. The exaggerated and arbitrary description of risk associated with having a travel history to Wuhan provided by China’s official statements and media reports may also worsen the public’s isolation of people with such an experience, despite it not being the original intention. Thus, social support and social policies should be available for potential patients, including the reporter of travel history, which would alleviate the social stigma attached to the travel history reporter and ease the discriminative atmosphere. For example, the objective, open and continuous epidemic information publicity and communication with the public based on reliable medical investigation and experience, as well as non-discriminatory treatment to the travel history reporter by epidemic prevention and control departments and staff may improve the situation. The government and media should also draw connections between regions and the infection with discretion to prevent the situation from worsening into regional discrimination. In addition, this study and past studies concur that the public fears restrictive epidemic control measures, such as quarantining [16–18]. On some level, this fear is an extension of the underlying anxiety surrounding an infectious disease with an unknown cause and possible fatal outcome [19]. Members of the public with a travel history would generally be subjected to the prevention and control process of quarantine after reporting, until the incubation period passes without incident. Fear of this has also become one of the factors that impact on the willingness to report. Therefore, effective health education strategies and public risk communication are necessary to allay such fear.

Third, this study showed that the members of the public that agreed that withholding travel history to high-risk epidemic regions would result in legal liability demonstrated a greater willingness to report (*P* < 0.05). To combat COVID-19, China implemented Class A (the highest level) epidemic prevention and control measures. Each province and city successively implemented first-level responses to the major public health emergency. During this time, all units and individuals were required to comply with epidemic-related measures enforced by disease prevention and control facilities and medical facilities, and truthfully provide relevant information. Deliberately withholding travel history to high-risk regions would result in a conviction for the crime of ‘endangering public security by dangerous means’, based on the severity of the outcome [20, 21]. Shanghai even introduced China’s first COVID-19 legislation: those who evaded quarantine by withholding their travel history would be diligently pursued for applicable legal liability based on the law [22]. Understanding that their action may result in serious consequences enforced by the legal system may suppress the public’s desire to withhold information. Thus, based on the existent legal framework on withholding travel history, the government should further expand public campaigns to explain legal obligations to report on travel history and emphasize the consequences of withholding information.

In investigating the cues that may encourage the public’s reporting of travel history, we found various types of effective cues, with the greatest number of respondents admitting to the effectiveness of hearing about cases where withholders of travel history were punished by law. This outcome could enhance the public’s acknowledgment of the illegality of withholding information, as well as prompt public consideration of law enforcement dynamics. However, this study was unable to provide a conclusion regarding the public’s sensitivity toward different penalties and frequency of hearing cases of people being prosecuted, as well as the threshold that affects their ultimate actions. This aspect would require further exploration in future. Seeing others showing potential symptoms of COVID-19 also encourages individuals to report their travel history, as withholding information increases the concern of negatively impacting others. Epidemiological analysis from the Chinese Center for Disease Control and Prevention shows that there are more than 1000 cluster COVID-19 cases in the country, 83% of them are family-based, or occurring in common gathering places, such as medical institutions, schools, shopping malls, and factories [23]. Another cue was the public speaking by communities. Thus far, grassroots communities, including rural communities, have played a unique role in China’s epidemic prevention and control as well as in social governance [24]. The power of grassroots leaders made it possible to manage residents of each jurisdiction systematically to monitor the epidemic’s advancement, promote epidemic information, and educate residents on healthy behavior. The community is the public’s direct recipient, processer, and promoter of travel history reports to high-risk epidemic regions, and as such, most members of the public report to their communities first if they have the willingness to report. China’s leader Xi Jinping has commented as follows: ‘The community is the frontline in epidemic prevention and control, and is the most effective line of defense in blocking external infectious sources and containing internal spread’ [25]. Under the current situation, where the epidemic is spreading globally, each country can consider mobilizing their communities for prevention and control as China has done—to place the power of prevention and control in the community.

Nevertheless, this study has the following limitations. First, the public’s willingness and beliefs associated with reporting on travel history to high-risk epidemic regions may change with the course of the epidemic. A cross-sectional study is limited in its usefulness in capturing this type of dynamic change. Future research may consider a longitudinal design. Second, this study only analyzed the effects that socio-demographic characteristics and beliefs associated with reporting travel history had on the willingness to report. There may be other factors affecting the public’s willingness to report. Third, considering the cost and convenience of conducting research, this study only selectively investigated a portion of provinces and cities based on the severity of the epidemic outbreak. For more universal results, a study on a larger scale may be necessary. Fourth, this study only measures and analyzes the respondent’s subjective social status. Future research may need to consider the impact of objective social economic status, which will contribute to more precise crowd intervention.

## Conclusions

Our study on the Chinese public’s willingness to report on travel history to high-risk epidemic regions showed an inclination to withhold travel history, albeit in a small proportion of the public. Considering that any incident of withheld history can result in unpredictable outcomes, this finding demands attention. Our study also indicated that the belief of the benefits of reporting, obstacles to reporting, and legal consequences of withholding travel history would affect willingness to report. Therefore, on the one hand, suitable public risk communication and public advocacy strategies should be carried out to reinforce the understanding that travel history reporting allows the individual and others to receive infection screening and treatment earlier, and alleviate the fear of potentially being quarantined after reporting. On the other hand, social support and social policies should be made available to potential patients, including the reporters of travel history, to eliminate the feeling of stigma that may arise from reporting travel history. The government and media should draw connections between regions and the infection with great discretion, to alleviate the phenomena of stigmatization and discrimination, to which potential patients are subjected. Finally, reinforcing the legal accountability of withholding travel history and strengthening systematic community monitoring are measures currently taken by China to promote reports on travel history to high-risk regions. These actions provide internationally relevant experiences to countries that are currently facing the spread of the epidemic and countries at risk of its potential spread in response to the WHO’s advocacy for non-pharmaceutical interventions.

## Supplementary information

**Additional file 1.** Questionnaire. Questionnaire developed for this research.

## Data Availability

The datasets generated and analyzed during the current study are not publicly available due to privacy restrictions. Respondents were informed during the consent process that the data they provide would be available only to the Harbin Medical University.

## Abbreviations

COVID-19: Coronavirus Disease 2019
SARS-CoV-2: Severe Acute Respiratory Syndrome Coronavirus 2
WHO: World Health Organization
