# Supplementary material for: Willingness and beliefs associated with reporting travel history to high-risk coronavirus disease 2019 epidemic regions among the Chinese public: a cross-sectional study
Source: BMC Public Health. 2020 Jul 25;20:1164. doi: 10.1186/s12889-020-09282-4 (PMC7382320; doi:10.1186/s12889-020-09282-4)
Supplement: Supplementary file 1 — Additional file 1. Questionnaire. Questionnaire developed for this research. [file 12889_2020_9282_MOESM1_ESM.docx]

**Additional file 1:**

**Questionnaire. Questionnaire developed for this research**

**A public survey on** **willingness and cognition toward** **reporting travel history to high-risk coronavirus disease 2019 epidemic regions**

***Thank you for participating in this survey on the willingness and cognition toward reporting travel history to high-risk coronavirus disease 2019 epidemic regions. This is an anonymous survey, and all information collected will be used for academic research with your consent. Please answer all questions truthfully. Thank you again for your participation.***

**A. Socio-demographic information**

**A 1** Gender: 🞏Male 🞏female

**A 2** Age: 🞏≤20 🞏21–30 🞏31–40 🞏41–50 🞏≥51

**A 3** Education: 🞏High school graduate and below 🞏Junior college

🞏College graduate 🞏Master degree and above

**A 4** What is your Marital status: 🞏 Single 🞏 Married 🞏 Separated

🞏Divorced 🞏 Widowed

**A 5** Place of residence: 🞏City 🞏Countryside

**A 6** Living arrangement: 🞏Living alone 🞏Living with family or friends 🞏Living with others

**A 7** Do you have a religion? 🞏Yes 🞏No

**A 8** Subjective social status

8.1 Please rate your social status in the country, and give a score from 1-10 points.

(10 points represents the people who are the best off - those who have the most money, the most education and the most respected jobs in their country; 1 point represents the people who are the worst off - who have the least money, least education, and the least respected jobs or no job in their country)

8.2 Please rate your social status in the community where you are, and give a score from 1-10 points.

(10 points represents the people who are the best off - those who have the most money, the most education and the most respected jobs in their community; 1 point represents the people who are the worst off - who have the least money, least education, and the least respected jobs or no job in their community)

**B. Willingness to report and cognition**

**B 1** Supposing you have a travel history to Wuhan, would you report this to the designated department, facility, or personnel?

🞏definitely would not report

🞏would not report

🞏unsure

🞏would report

🞏definitely would report

**B 2** Please choose your level of approval for the following statements.

|  | strongly disagree | disagree | unsure | agree | strongly agree |
| --- | --- | --- | --- | --- | --- |
| Travel history has an impact on contracting COVID-19 | 🞏 | 🞏 | 🞏 | 🞏 | 🞏 |
| Reporting can confirm whether I contracted COVID-19 earlier | 🞏 | 🞏 | 🞏 | 🞏 | 🞏 |
| Reporting can help discover earlier potential patients infected by coming in contact with me | 🞏 | 🞏 | 🞏 | 🞏 | 🞏 |
| Reporting makes me feel stigma | 🞏 | 🞏 | 🞏 | 🞏 | 🞏 |
| Reporting would lead to great expenses for follow-up tests or treatment | 🞏 | 🞏 | 🞏 | 🞏 | 🞏 |
| I don’t know how to submit a report | 🞏 | 🞏 | 🞏 | 🞏 | 🞏 |
| Reporting is very inconvenient | 🞏 | 🞏 | 🞏 | 🞏 | 🞏 |
| I’m afraid that reporting may lead to my quarantine | 🞏 | 🞏 | 🞏 | 🞏 | 🞏 |
| Withholding travel history to high-risk epidemic regions will result in legal liability | 🞏 | 🞏 | 🞏 | 🞏 | 🞏 |

**C. Cues that promote reporting**

**C 1** Please select the cues that you think will prompt you to report travel history to high-risk coronavirus disease 2019 epidemic regions. (Multiple choice)

🞏Hearing about cases where withholders of travel history were punished by law

🞏People around me showing potential symptoms of COVID-19

🞏Advocacy on television, the internet, and other media

🞏Community public speaking

🞏Emergence or worsening of potential COVID-19 symptoms in myself

🞏Persuasion by family or friends

🞏Having a history of severe respiratory system conditions (e.g. SARS)
